# MicroRNA‐29b‐3p suppresses oral squamous cell carcinoma cell migration and invasion via IL32/AKT signalling pathway

**DOI:** 10.1111/jcmm.14794

**Published:** 2019-11-03

**Authors:** Jianya He, Wen Ye, Ni Kou, Kang Chen, Bai Cui, Xiaohong Zhang, Shuhai Hu, Tingjiao Liu, Lan Kang, Xiaojie Li

**Affiliations:** ^1^ Department of Prosthodontics College of Stomatology Dalian Medical University Dalian China; ^2^ Institute for Regenerative Medicine Shanghai East Hospital Shanghai Key Laboratory of Signaling and Disease Research School of Life Sciences and Technology Tongji University Shanghai China; ^3^ Institute of Cancer Stem Cell Dalian Medical University Dalian China; ^4^ Department of Oral and Maxillofacial Surgery College of Stomatology Dalian Medical University Dalian China; ^5^ Department of Oral Pathology College of Stomatology Dalian Medical University Dalian China

**Keywords:** AKT, IL32, migration and invasion, miR‐29b‐3p, OSCC

## Abstract

Oral squamous cell carcinoma (OSCC) is aggressive accompanied with poor prognosis. We previously isolated the most invasive cells resembling the invasive tumour front by microfluidic technology and explored their differentially expressed microRNAs (miRNAs) in our previous work. Here, we verified the miR‐29b‐3p as a guarder that suppressed migration and invasion of OSCC cells and was down‐regulated in the most invasive cells. Besides that, the invasion suppression role of miR‐29b‐3p was achieved through the IL32/AKT pathway. Thus, miR‐29b‐3p and IL32 might serve as therapeutic targets for blocking the progression and improving the outcome of OSCC.

## 1 INTRODUCTION

Oral squamous cell carcinoma (OSCC) is the most common malignant tumour of the oral and maxillofacial region.^1^ Oral squamous cell carcinoma is aggressive and readily metastasizes to regional lymph nodes, which is associated with poor prognosis.^2^ Invasion is the first step in metastasis. Thus, clarifying the mechanism underlying OSCC invasion and metastasis is critical for the development of new therapeutic strategies for improving patient outcome.

The invasion tumour front, which consists of 3‐6 layers of tumour cells or cell clusters at the forefront of the tumour‐host junction, harbours the most invasive cells in the tumour and is responsible for tumour cell dissemination.^3^ Limited to the technique for the identification and isolation of the invasion tumour front, it is difficult to explore the molecular mechanisms governing their behaviour. In the previous work, we isolated the most invasive cells which resembled the invasive tumour front by microfluidic technology from OSCC cell line. These invasion tumour front–like cells serve as a good model for exploring the underlying mechanism for OSCC invasion behaviour.^4^

MicroRNAs (miRNAs) participate in many aspects of tumour properties by regulating gene expression post‐transcriptionally.^5^, ^6^ Recent studies have shown that miRNAs play an essential role in the transformation of oral leukoplakia to OSCC and can serve as biomarkers for this process.^7^ They also regulate essential genes involved in OSCC invasion and metastasis at the post‐transcriptional level.^8^, ^9^ We explored miRNA profile in OSCC cell lines in our previous work and characterized some miRNAs with differential expression between the cell lines with high and low invasion ability.^4^ Among these miRNAs, miR‐29b‐3p is significantly down‐regulated in highly invasive OSCC cell line.

MiR‐29b had antitumour effects in various tumours and is down‐regulated in pancreatic cancer cells,^10^ colorectal cancer^11^ and human glioblastoma multiforme.^12^ MiR‐29b acts as a tumour suppressor in tongue squamous cell carcinoma by inhibiting the expression of genes involved in tumour cell proliferation, migration and invasion by targeting specificity protein 1 and thereby altering phosphatase and tensin homolog/AKT signalling.^13^ However, there is little information on the function and mechanism of miR‐29b in OSCC invasion.

In the present study, we show that miR‐29b‐3p is down‐regulated in OSCC invasive cells and inhibits OSCC cell migration and invasion by suppressing the interleukin (IL) 32/AKT pathway.

## 2 MATERIALS AND METHODS

### 2.1 Cell culture

The human UM‐SCC6 cell line was a kind gift from Peking Union Medical University. The OSCC invasion tumour front–like cells termed as UM‐SCC6‐M were separated from UM‐SCC6 cells via the microfluidic chip in vitro.^4^ They were cultured in DMEM/HG (HyClone) medium, supplemented with 10% foetal bovine serum (FBS) (Sciencell) and 1% penicillin streptomycin (Hyclone).

### 2.2 Wound healing assay

Cell migration capacity was validated by wound healing assay. The cells were trypsinized and seeded into six‐well plates (5 × 10^5^ cells per well). When the cells grew to 90% confluence, the monolayers of cells were scratched by a sterile 200 µL pipette tip and washed with PBS for three times to remove cellular debris and then allowed to culture in serum‐free medium. The wound area at the start and end of each experiment was recorded by inverted microscope (Olympus IX 71) and calculated with Image‐Pro Plus 6.0 at 0, 12, 24 and 36 hours after scratch. The cell migration rate was calculated by the decreased wound width in the wound area normalized to the initial wound width. The data represent the mean ± standard error of three independent experiments.

### 2.3 Transwell invasion assay

Cell invasion ability was assessed by transwell plates (Corning). Invasion inserts with 8µm pore were coated with 80 µL Matrigel™ (Corning) and dried overnight at 37°C incubator. About 3 × 10^4^ cells in 200 µL serum‐free medium were seeded into the top chambers, and 600 µL medium supplemented with 20% serum was used as a chemoattractant in the lower chamber. After incubating for 24 hours at 37°C in 5% CO_2_, the medium in top chambers was removed, and the cells were fixed with 4% paraformaldehyde and stained with 0.1% crystal violet. The non‐invading cells on the upper sides of membrane were removed by cotton wool. The cells on the lower sides of membrane were counted. Five random fields (×200) were selected and calculated the average. The data represent the mean ± standard error of three independent experiments.

### 2.4 Total RNA isolation and Quantitative PCR (qPCR)

Total RNA was isolated from the cells using TRIzol reagent (Invitrogen) according to the manufacturer's instructions. For mRNA analyses, first­strand cDNA was synthesized by 5X All‐In‐One RT Master Mix (ABMgood). qPCR was performed using KAPA SYBR^®^ FAST Universal qPCR Kits (KAPA Biosystems). Relative quantitation was calculated based on the 2^−ΔΔCt^ method. Gapdh was used as internal reference. For miRNA analyses, the mature miRNA was reverse transcribed with specific primers by Prime Script™ RT Master Mix (Takara). Then, qPCR was performed and analysed the same as for mRNA. U6 was used as internal reference. All reactions were carried out in triplicate. The primers used in this work were purchased from RiboBio.

### 2.5 The gene overexpression and RNA interference

Plasmids pcDNA6 and pLKO‐tet‐on were used to overexpressing and knocking down the IL32, respectively. The sequences of shRNA targeting human IL32 were shIL32‐1: TGGGGAGAGCTTTTGTGACAA; shIL32‐2: AGAGCTGGAGGACGACTTCAA; shIL32‐3: GCTCTGAACCCCAATCCTCAA. The mimic and the inhibitor of miR‐29b‐3p were purchased from Gene Pharma. The plasmids, mimic or inhibitor was transfected into the OSCC cell lines using Lipofectamine 2000 (Invitrogen) according to the manufacturer's instructions. Cells were harvested for analysis 48 hours later. All experiments were performed in triplicates.

### 2.6 Western blot analysis

Total proteins were extracted from cells with RIPA buffer (Sigma Aldrich), separated by SDS‐PAGE gels and transferred to polyvinylidene difluoride (PVDF) membrane (Millipore). The membranes were blocked with 5% nonfat milk and incubated with primary antibodies (β‐CATENIN, AKT, pAKT, mTOR, pmTOR, MEK, pMEK (Cell Signaling Technology), MMP2, SNAI1 (Abcam, Cambridge, UK), IL32, TWIST1, GAPDH (Proteintech) overnight at 4°C, followed by incubation with secondary antibodies for 1 hour at room temperature. The immunoreactive protein bands were detected by ECL detection system. GAPDH was used as a loading control.

### 2.7 mRNA sequencing and data analysis

An RNA‐seq library was prepared using the protocol of KAPA Stranded mRNA‐Seq Kit (Kapa Biosystems) for the Illumina platform, following the manufacturer's instructions. Paired‐end 150‐bp sequencing was performed on HiSeq 2500 system in Berry Genomics Company. All reads were aligned to the human reference genome hg38 from UCSC using TopHat. Only unique mapped reads were used for further analyses. Cufflinks was used to measure the relative abundance of each transcript using the Ensemble Transcript ID. Gene functional enrichment analysis was performed using Gene Set Enrichment Analysis (GSEA) (http://software.broadinstitute.org/gsea/index.jsp).

### 2.8 Statistical analysis

Statistical analysis was performed using SPSS. Student's *t* test was used to confirm comparisons of binary variables. Statistical significance was defined as *P* < .05.

## 3 RESULTS

### 3.1 MiR‐29b‐3p suppressed OSCC cell migration and invasion

**Figure 1:**
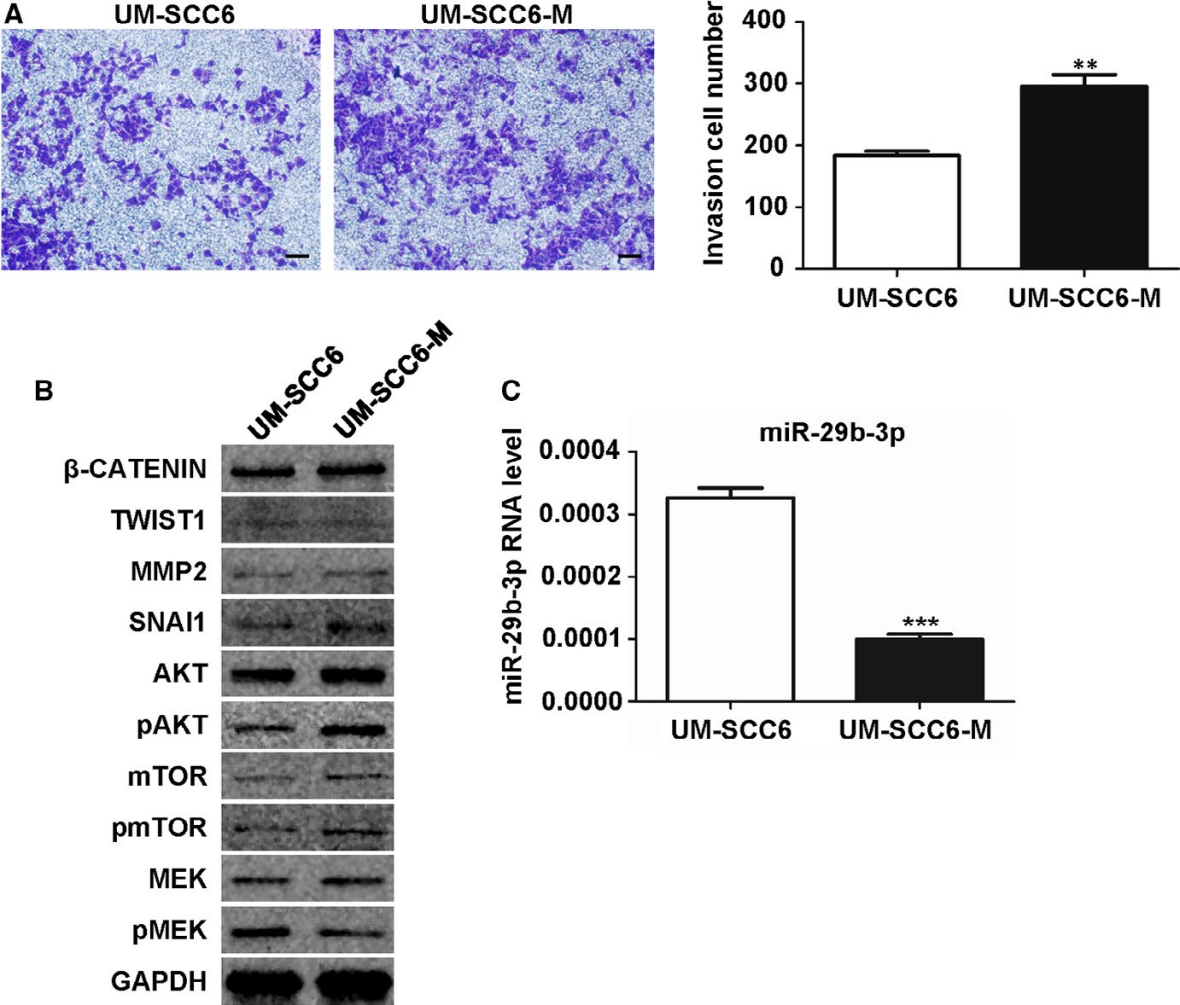
MiR‐29b‐3p showed low expression level in high invasive cell line UM‐SCC6‐M. A, Transwell invasion assay for UM‐SCC6 and UM‐SCC6‐M cells. B, Western blot of migration/invasion biomarkers' expression in UM‐SCC6 and UM‐SCC6‐M cells. C, qPCR analysis of RNA level of miR‐29b‐3p in UM‐SCC6 and UM‐SCC6‐M cells. Data represent the mean ± standard error of three independent experiments. *, *P* < .05; **, *P* < .01; ***, *P* < .001; ns, not significant

In our previous work, using microfluidic chip, we isolated invasion tumour front–like cells from OSCC cell line UM‐SCC6. The invasion tumour front–like cells, which we named UM‐SCC6‐M, exhibited mesenchymal properties and enhanced invasiveness.^4^ The UM‐SCC6 cells were interconnected and exhibited a cobblestone organization, whereas UM‐SCC6‐M cells were spindle‐shaped and loosely connected (Figure S1A). The UM‐SCC6‐M cells also showed higher migratory capacity **(**Figure S1B) and invasiveness (Figure 1A) than UM‐SCC6 cells. Then, we examined the migration and invasion related markers and found that the protein level of SNAI1 and AKT was increased in UM‐SCC6‐M cells, with pAKT showing the greatest augment (Figure 1B).

**Figure 2:**
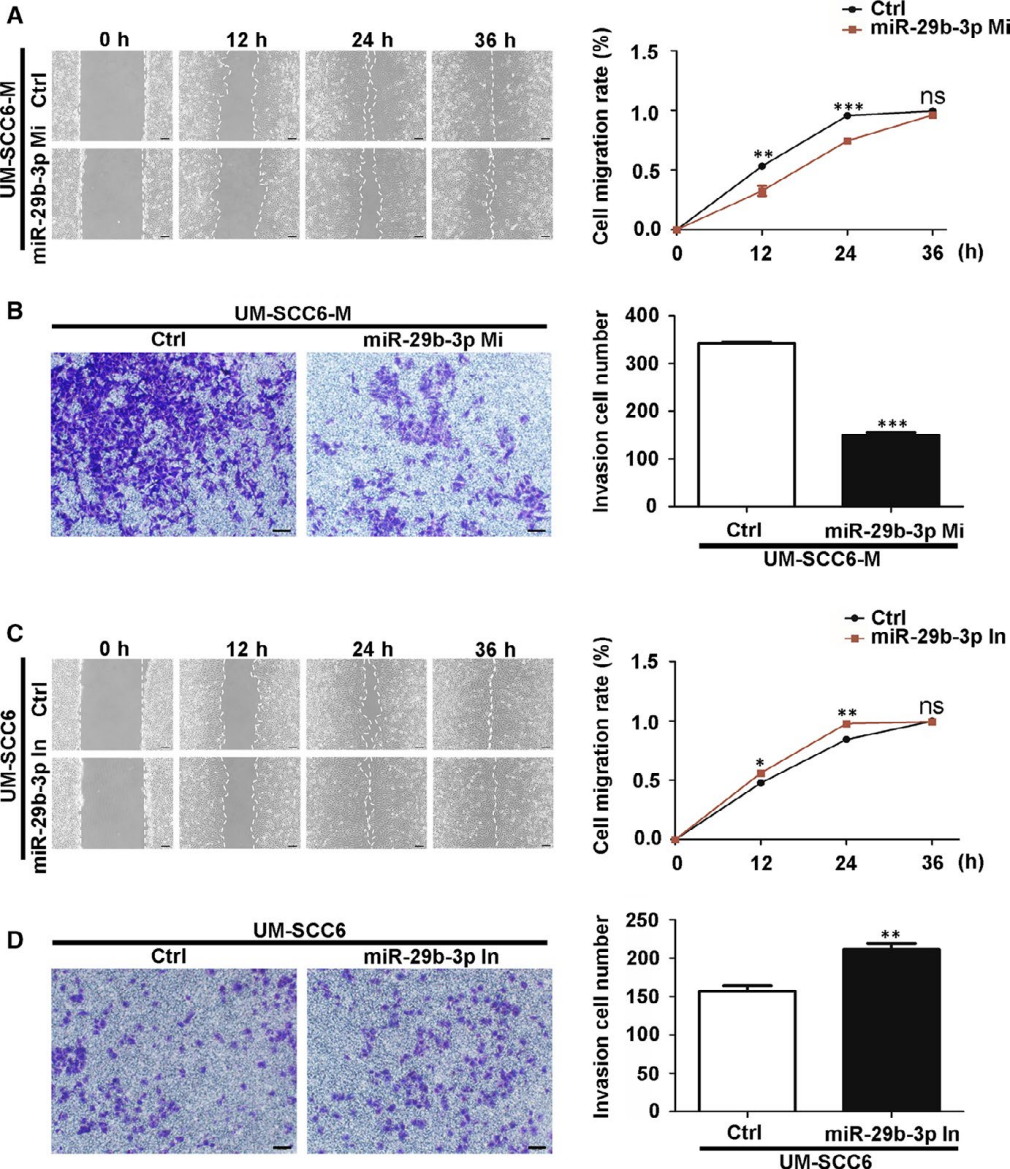
MiR‐29b‐3p suppressed OSCC cell migration and invasion. A, B, Effect of miR‐29b‐3p mimic on UM‐SCC6‐M cells' migration (A) and invasion (B), as evaluated with wound healing and transwell invasion assays, respectively. C, D, Effect of miR‐29b‐3p inhibitor on UM‐SCC6‐M cells' migration (C) and invasion (D), as evaluated with wound healing and transwell invasion assays, respectively. Scale Bar = 100 μm. Data represent the mean ± standard error of three independent experiments. *, *P* < .05; **P < .01; ***, *P* < .001; ns, *P* > .05. Ctrl, control; In, inhibitor; Mi, mimic

The small RNA sequencing data from our previous work showed that miR‐29b‐3p was significantly down‐regulated in UM‐SCC6‐M cells, which was supported by qPCR (Figure 1C). To explore the function of miR‐29b‐3p in OSCC cells, we employed the inhibitor and mimic of miR‐29b‐3p (Figure S2). The results of the wound healing and transwell invasion assays showed that transfection of the miR‐29b‐3p mimic markedly suppressed both migration (Figure 2A) and invasion (Figure 2B) in UM‐SCC6‐M cells, whereas inhibiting miR‐29b‐3p in UM‐SCC6 cells by inhibitor enhanced these behaviours (Figure 2C,D).

### 3.2 AKT signalling mediated the migration and invasion of OSCC cells

**Figure 3:**
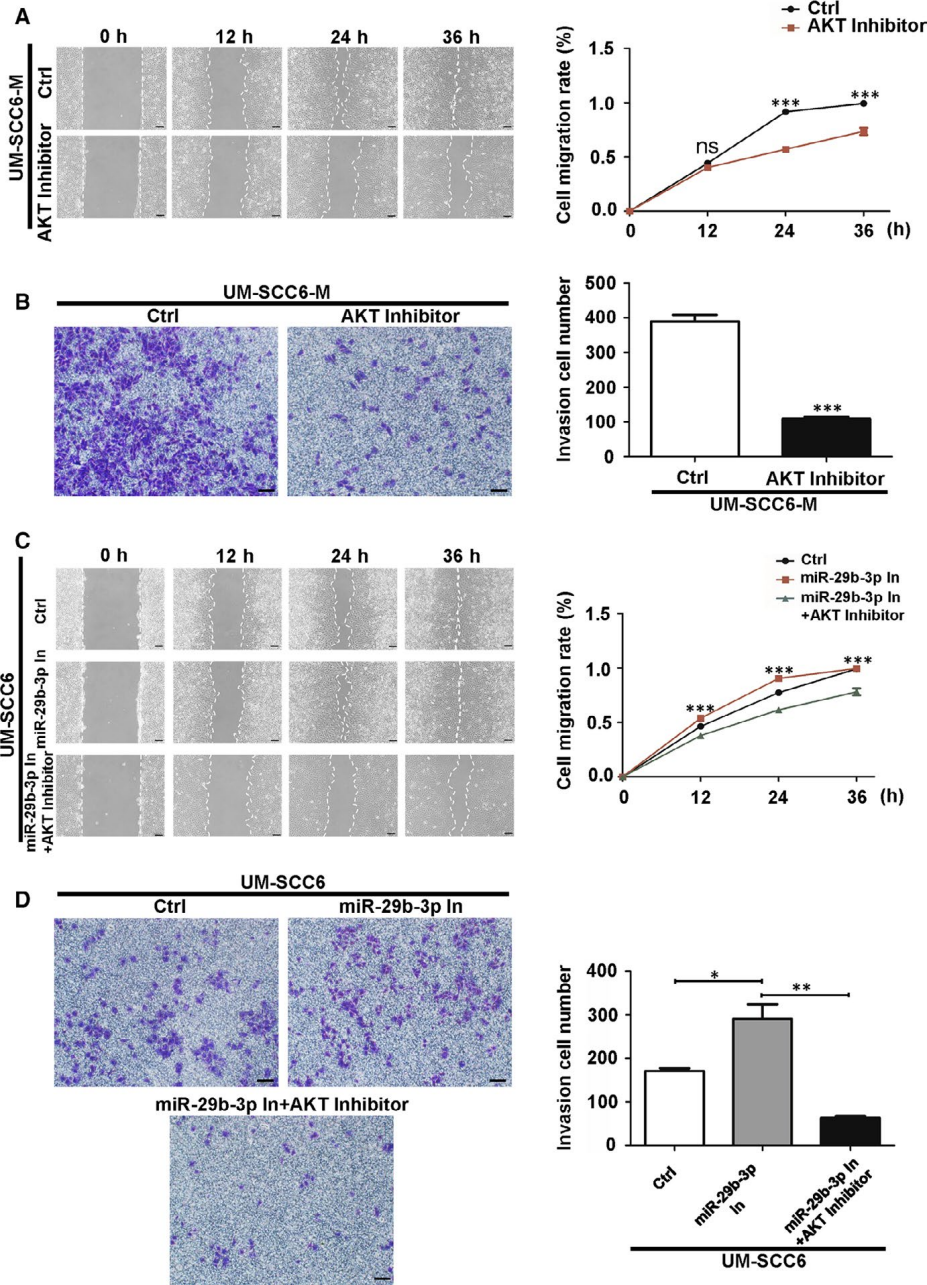
AKT signalling mediated the migration and invasion of OSCC cells. A, B, Effect of MK‐2206 on UM‐SCC6‐M cell migration (A) and invasion (B), as evaluated with wound healing and transwell invasion assays, respectively. C, D, Effect of AKT inhibitor MK‐2206 on the migration (C) and invasion (D) of UM‐SCC6 cells transfected with miR‐29b‐3p inhibitor as evaluated with wound healing and transwell invasion assays, respectively. Scale Bar = 100 μm. Data represent the mean ± standard error of three independent experiments. *, *P* < .05; **, *P* < .01; ***, *P* < .001; ns, not significant

The AKT pathway is widely acknowledged as a critical regulator of tumour progression.^14^ To determine whether the behaviours of UM‐SCC6‐M cells were due to the up‐regulation of pAKT, cells were treated with the AKT inhibitor, MK‐2206. We found that the treatment suppressed cells' migration and invasion (Figure 3A,B). The same effect was also observed in UM‐SCC6 cells, which exhibited only weak migratory and invasive capacities and MK‐2206 made them further weaker (Figure S3A,B). Meanwhile, MK‐2206 attenuated the migrating and invading stimulatory effect of miR‐29b‐3p inhibitor in UM‐SCC6 cells (Figure 3C,D). These results demonstrate that AKT signalling mediates the migration and invasion of OSCC cells and may be a downstream effector of miR‐29b‐3p.

### 3.3 IL32 was a downstream target of miR‐29b‐3p

To clarify the mechanism underlying the effects of miR‐29b‐3p, we carried out transcriptional profiling of UM‐SCC6 and UM‐SCC6‐M cells by second‐generation mRNA deep sequencing. After removing those genes expressed at extremely low levels (fragments per kilobase of transcript per million mapped reads (FPKM) < 5 in all samples), we obtained 10,721 genes (Figure S4A). Most of which were expressed at similar levels in the two cell lines (*R* ^2^ = 0.9336). The Kyoto Encyclopedia of Genes and Genomes (KEGG) analysis showed that the 569 genes up‐regulated in UM‐SCC6 cells were enriched in tumour‐related pathways (fold change (FC) > 1.5) (Figure S4B), whereas the 295 UM‐SCC6‐M cell‐specific genes were enriched in tumour metastasis‐related pathways (FC > 1.5) (Figure S4C), such as those associated with the cytoskeleton and cell adhesion.

We next compared the transcriptional profiles of UM‐SCC6‐M cells transfected with miR‐29b‐3p mimic or a negative control. The analysis of the 10 535 expressed genes (Figure S4D) showed that miR‐29b‐3p mimic did not cause significant changes in transcription (*R* ^2^ = 0.9473). The 402 up‐regulated genes in UM‐SCC6‐M cells transfected with miR‐29b‐3p mimic were enriched in tumour‐related and metabolic pathways (FC > 1.5) (Figure S4E), whereas the 319 down‐regulated genes were enriched in tumour metastasis‐related pathways (FC > 1.5) (Figure S4F), including those associated with the extracellular matrix, cytoskeleton and cell adhesion, which was consistent with the results of the functional assays.

**Figure 4:**
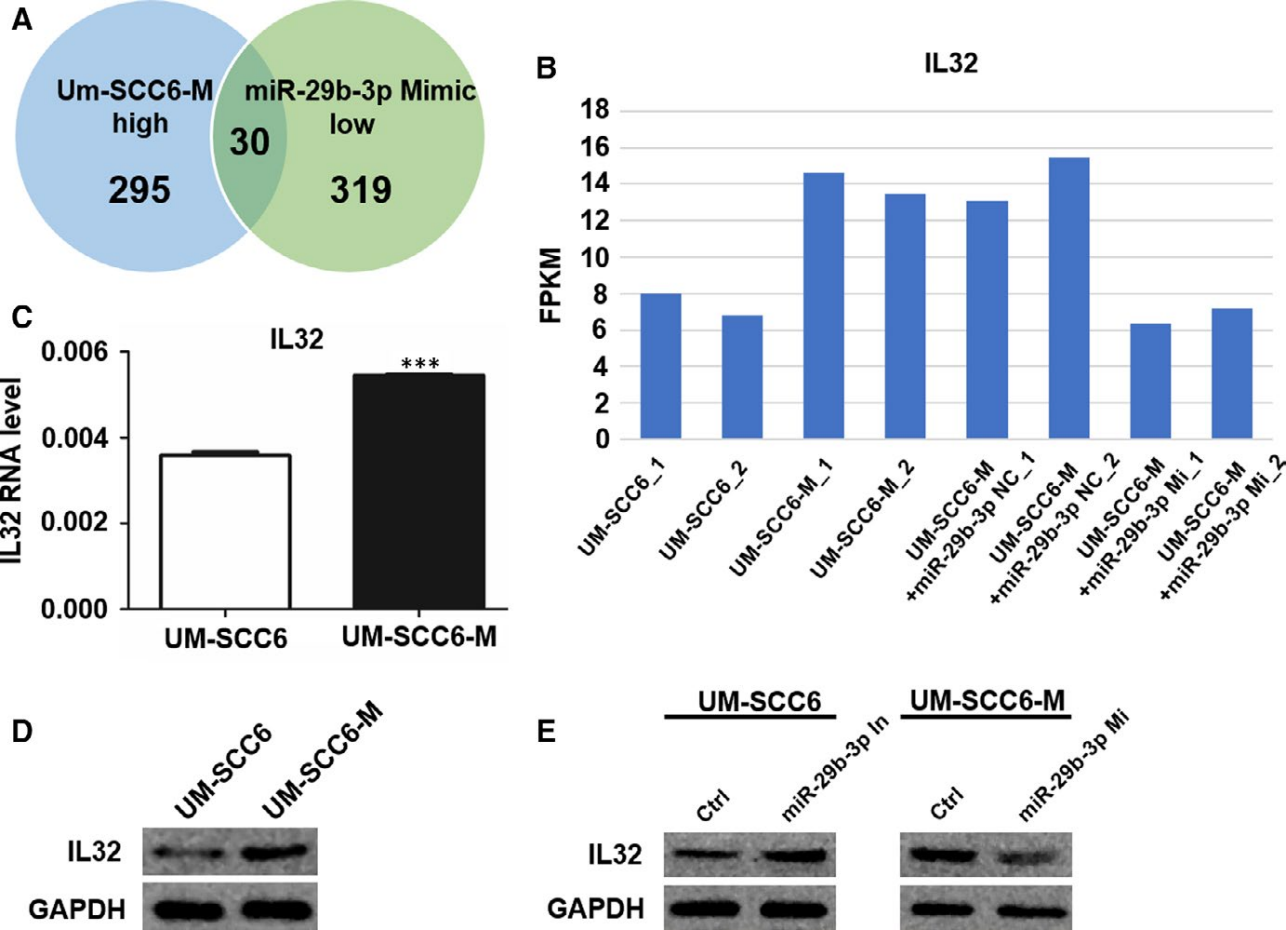
IL32 was a downstream target of miR‐29b‐3p. A, Venn diagram shows the genes with high expression in UM‐SCC6‐M cells and down‐regulated by miR‐29b‐3p mimic. B, FPKM of IL32 in the mRNA sequencing data. C, D, mRNA (C) and protein (D) level of IL32 in UM‐SCC6 and UM‐SCC6‐M cells. E, Western blot showed the effects of miR‐29b‐3p mimic and inhibitor on IL32. Data represent the mean ± standard error of three independent experiments. ***, *P* < .001

Then, we explored the targets of miR‐29b‐3p which could be involved in the regulation of metastasis. Since miR‐29b‐3p was down‐regulated in UM‐SCC6‐M cells and miRNAs typically act as negative regulators of gene expression, we focused on the 30 genes which were both enriched in UM‐SCC6‐M compared with UM‐SCC6 and decreased by miR‐29b‐3p mimic infection (Figure 4A). We compared these 30 genes with the 842 targets of miR‐29b‐3p predicted using miRanda. Then, we got IL32 which was the only candidate both in the 30 genes list and in the 842 candidate targets of miR‐29b‐3p. So we focused on IL32 to conduct further research. In accordance with the mRNA sequencing data (Figure 4B), qPCR and Western blotting showed that IL32 was up‐regulated in UM‐SCC6‐M cells at the mRNA and protein levels (Figure 4C,D). More importantly, it has been reported that miR‐29b regulated IL32 via direct binding to the 3′ untranslated region of the *IL‐32* transcript.^15^ Furthermore, by transfecting UM‐SCC6 and UM‐SCC6‐M cells with the miR‐29b‐3p inhibitor and mimic, respectively, we found that IL32 was under the negative regulation by miR‐29b‐3p at the post‐transcriptional level (Figures 4E and S5).

### 3.4 IL32 promoted OSCC cell migration and invasion by activating AKT signalling

**Figure 5:**
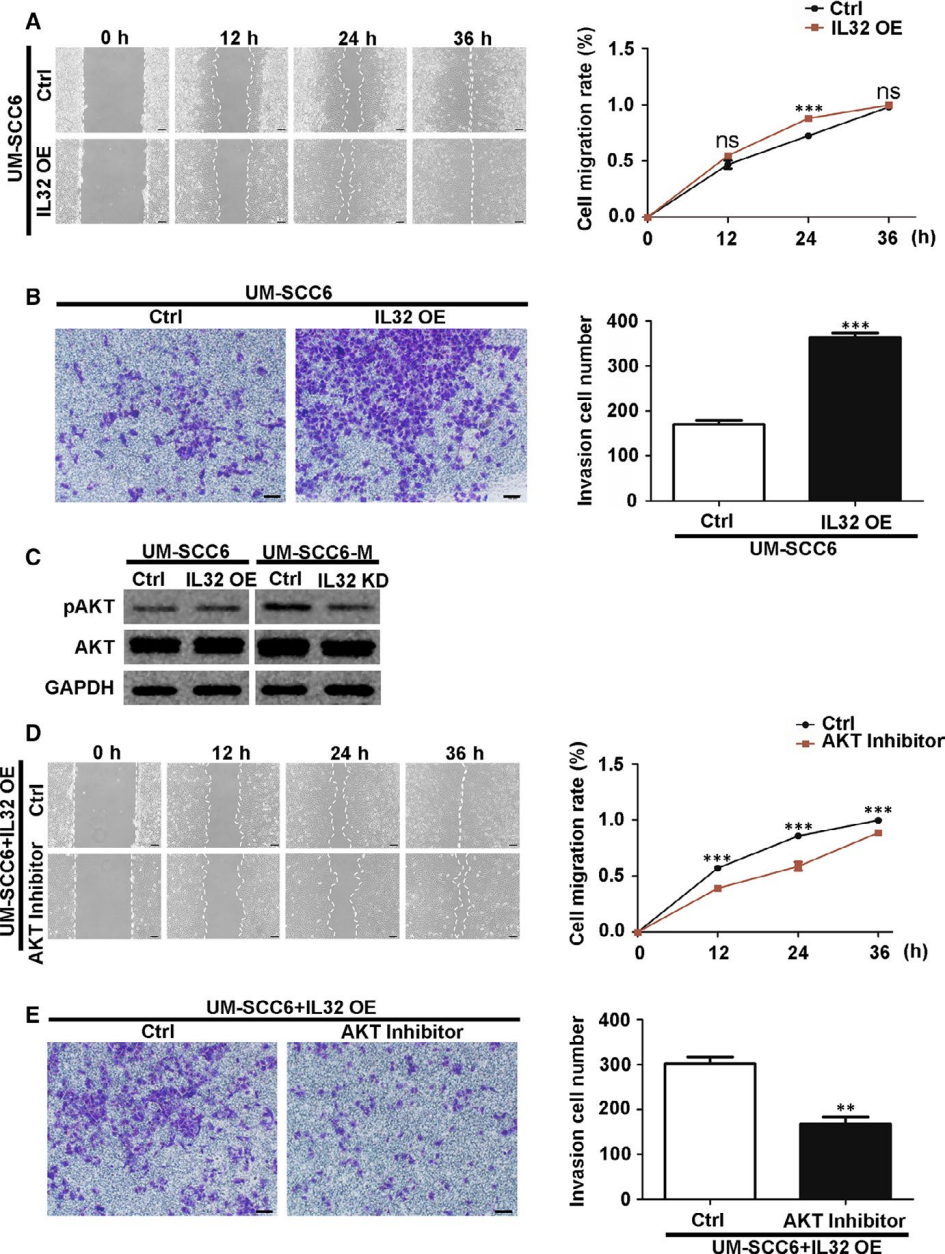
IL32 promoted OSCC cell migration and invasion by activating AKT signalling. A, B, Effect of IL32 overexpression and knockdown on UM‐SCC6 cells' migration (A) and invasion (B), as evaluated with wound healing and transwell invasion assays, respectively. C, Effect of IL32 overexpression and knockdown on protein level of AKT and pAKT. D, E, Effect of AKT inhibitor MK‐2206 on the migration (D) and invasion (E) of UM‐SCC6 cells with IL32 overexpressed, as evaluated with wound healing and transwell invasion assays, respectively. Scale Bar = 100 μm. Data represent the mean ± standard error of three independent experiments. **, *P* < .01; ***, *P* < .001; KD, knockdown; ns, not significant; OE, overexpression

IL32 is a cytokine involved in cancer development.^16^ It was reported to promote breast cancer cell invasion and metastasis via integrin β3/p38 mitogen‐activated protein kinase (MAPK) signalling.^17^ We therefore investigated the role of IL32 in OSCC migration and invasion and found that overexpression of IL32 in UM‐SCC6 cells stimulated both of these behaviours (Figure 5A,B and S6A). On the contrary, migration and invasion of UM‐SCC6‐M cells were suppressed by IL32 knockdown (Figure S6A‐C).

IL32 was previously reported to enhance metastatic potential in gastric cancer by activating AKT signalling.^18^ So IL32 might be the reason for the activated AKT in UM‐SCC6‐M cells. As expected, overexpression and knockdown of IL32 altered AKT phosphorylation (Figure 5C). Additionally, AKT inhibition completely abolished the stimulatory effect of IL32 on the migration and invasion of UM‐SCC6 cells (Figure 5D,E).

### 3.5 MiR‐29b‐3p suppressed migration and invasion via the IL32/AKT pathway

**Figure 6:**
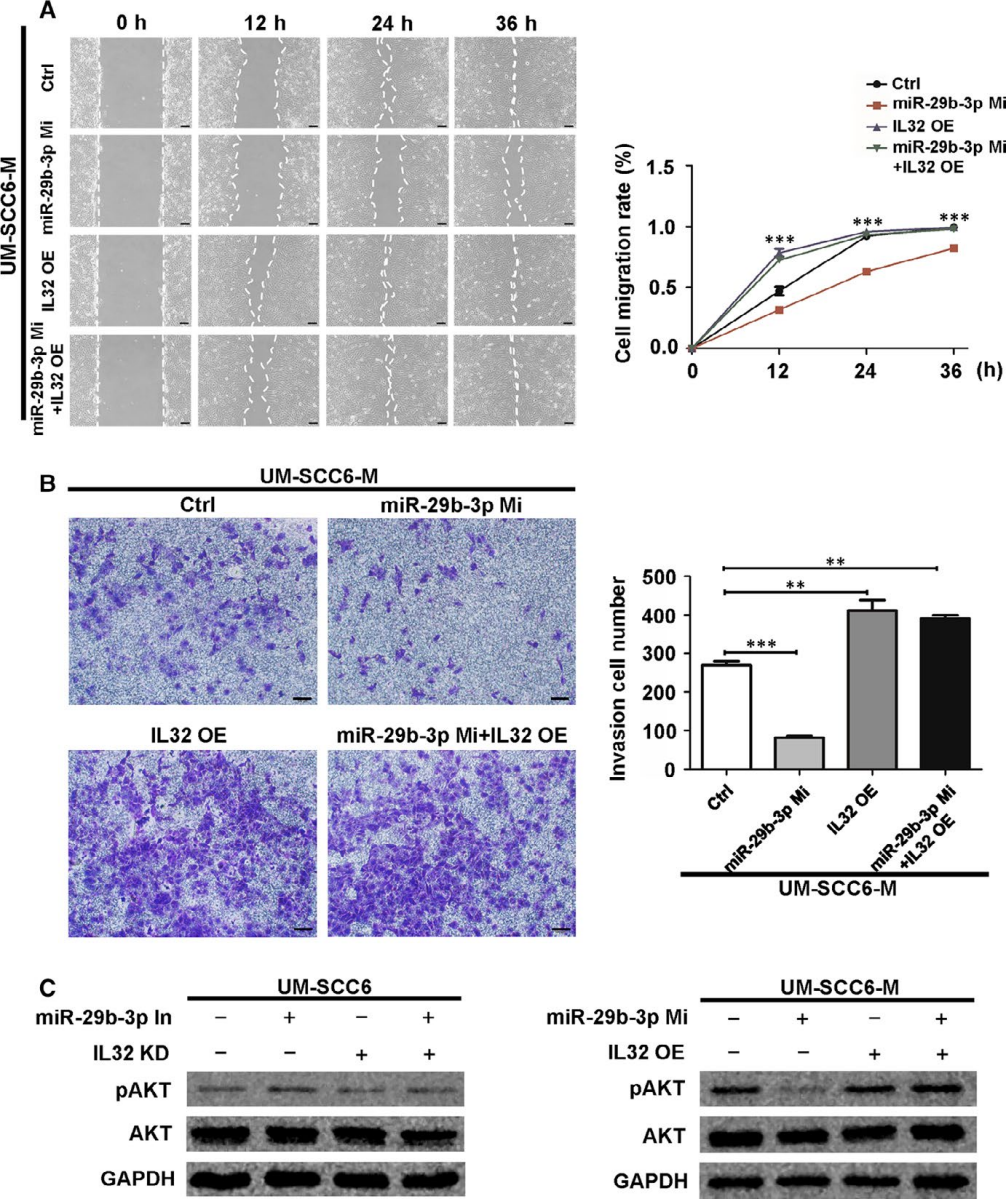
MiR‐29b‐3p suppressed migration and invasion via the IL32/AKT pathway. A, B, Effect of IL32 overexpression on the migration (A) and invasion (B) of UM‐SCC6‐M cells with miR‐29b‐3p mimic transfected, as evaluated with wound healing and transwell invasion assays, respectively. C, Western blot showed the AKT and pAKT level under the effect of IL32 and miR‐29b‐3p. Scale Bar = 100 μm. Data represent the mean ± standard error of three independent experiments. **, *P* < .01; ***, *P* < .001. Ctrl, control; In, inhibitor; KD, knockdown; Mi, mimic; OE, overexpression

The above results implied that IL32 mediated the effects of miR‐29b‐3p on AKT signalling. Indeed, miR‐29b‐3p mimic interfered with the migration and invasion of UM‐SCC6‐M cells, overexpression of IL32 abrogated its effects and restored the migratory and invasive potentials of the cells (Figure 6A,B). Conversely, knockdown of IL32 reversed the effect of the miR‐29b‐3p inhibitor on migration and invasion of UM‐SCC6 cells (Figure S7A,B). A similar effect was observed for AKT phosphorylation level, which was found to be regulated by the miR‐29b‐3p/IL32 axis (Figure 6C). As expected, the AKT inhibitor MK‐2206 suppressed the migration and invasion of UM‐SCC6 cells transfected with the miR‐29b‐3p mimic and IL32 overexpression plasmid (Figure S7C,D). Taken together, miR‐29b‐3p suppresses migration and invasion of OSCC via the IL32/AKT axis.

## 4 DISCUSSION

Oral squamous cell carcinoma is aggressive and readily metastasizes to regional lymph nodes, which is associated with poor prognosis and a low survival rate. Although the pathogenesis of OSCC is not fully understood, invasion tumour front cells can, to some extent, explain the biological properties of OSCC.^19^ In the present work, we took the advantage of the invasion tumour front–like cells (UM‐SCC6‐M cells) we previously established, which exhibited enhanced migratory and invasive capacity. We deeply explored the function and mechanism of miR‐29b‐3p, which is significantly down‐regulated in UM‐SCC6‐M cells in our previous miRNA sequencing data. And we found that miR‐29b‐3p suppresses migration and invasion of OSCC via the IL32/AKT axis.

MiR‐29b is known to be a critical suppressor in tumour progression, regulating epigenetics, proliferation, apoptosis, differentiation, metastasis and chemosensitivity.^20^ MiR‐29b‐3p is a member of the miR‐29 family, it has been reported as an antitumour effector in colorectal cancer,^21^ glioblastoma,^12^ gastric cancer,^22^ breast cancer^23^ and tongue squamous cell carcinoma,^13^ and our present study provided new evidence of the important role of miR‐29b‐3p in OSCC invasion.

By transcription profiling and miRNA targets analysis, we found out IL32, as a downstream target of miR‐29b‐3p. IL32 is implicated in the progression of a variety of malignancies including colorectal cancer,^24^ breast cancer,^25^, ^26^ head and neck squamous cell carcinoma,^27^ lung adenocarcinoma^28^ and gastric cancer.^18^ It was reported that miR‐29b regulates IL32 via direct binding to the 3′ untranslated region of the *IL‐32* transcript in hepatitis B virus infection.^15^ As an inflammatory cytokine, IL32 is linked to multiple tumour pathways including NF‐κB,^28^ VEGF/STAT3^26^ and p38 MAPK.^29^ In the present study, we showed that IL32 acts as a mediator between miR‐29b and the AKT pathway in the regulation of OSCC cells migration and invasion. This is also supported by the observation that IL32 induces the expression of AKT in osteoclasts^30^ and gastric cancer.^18^

In conclusion, the present study demonstrated that miR‐29b‐3p played an antitumour role in the migration and invasion of OSCC cells via suppressing the IL32/AKT signalling axis. These findings provide new insight into the mechanistic basis for OSCC metastasis and suggest that miR‐29b‐3p‐based treatment may enable promising new strategies to overcome OSCC metastasis.

## CONFLICT OF INTEREST

The authors confirm that there are no conflicts of interest.

## AUTHOR CONTRIBUTIONS

X. Li, L. Kang and T. Liu designed the experiments and wrote the manuscript. J. He, W. Ye and N. Kou carried out the experiments. K. Chen and B. Cui provided the technical support. X. Zhang and S. Hu performed the data analysis and revised the manuscript. All the authors read and approved the final manuscript.

## Data Availability

The data that support the findings of this study are available from the corresponding author upon reasonable request.
