# Supplementary figures and images for: MicroRNA‐29b‐3p suppresses oral squamous cell carcinoma cell migration and invasion via IL32/AKT signalling pathway
Source: J Cell Mol Med. 2019 Nov 3;24(1):841–9. doi: 10.1111/jcmm.14794 (PMC6933408; doi:10.1111/jcmm.14794)

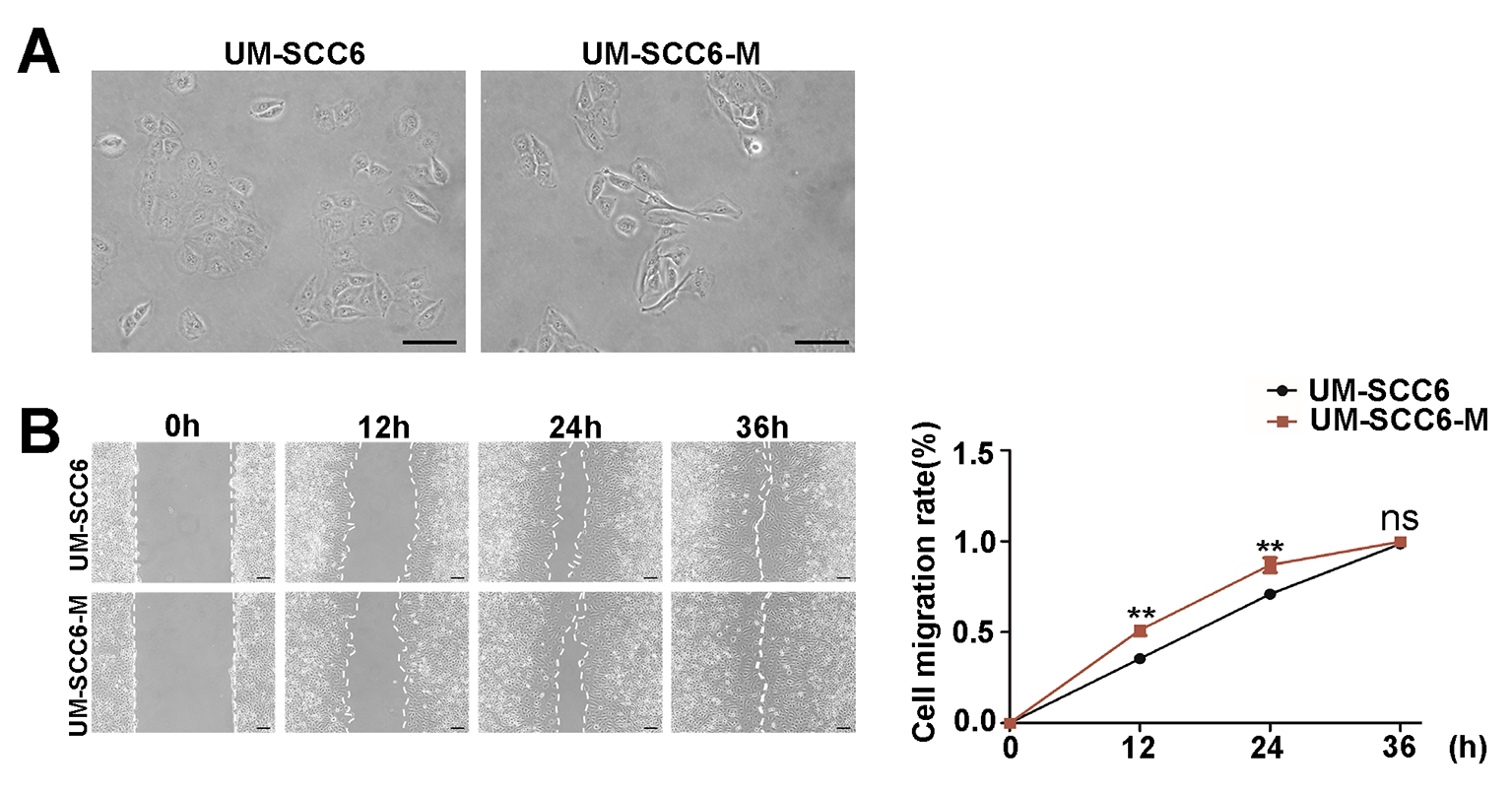

Supplement: Supplementary file 1 [file JCMM-24-841-s001.tif]

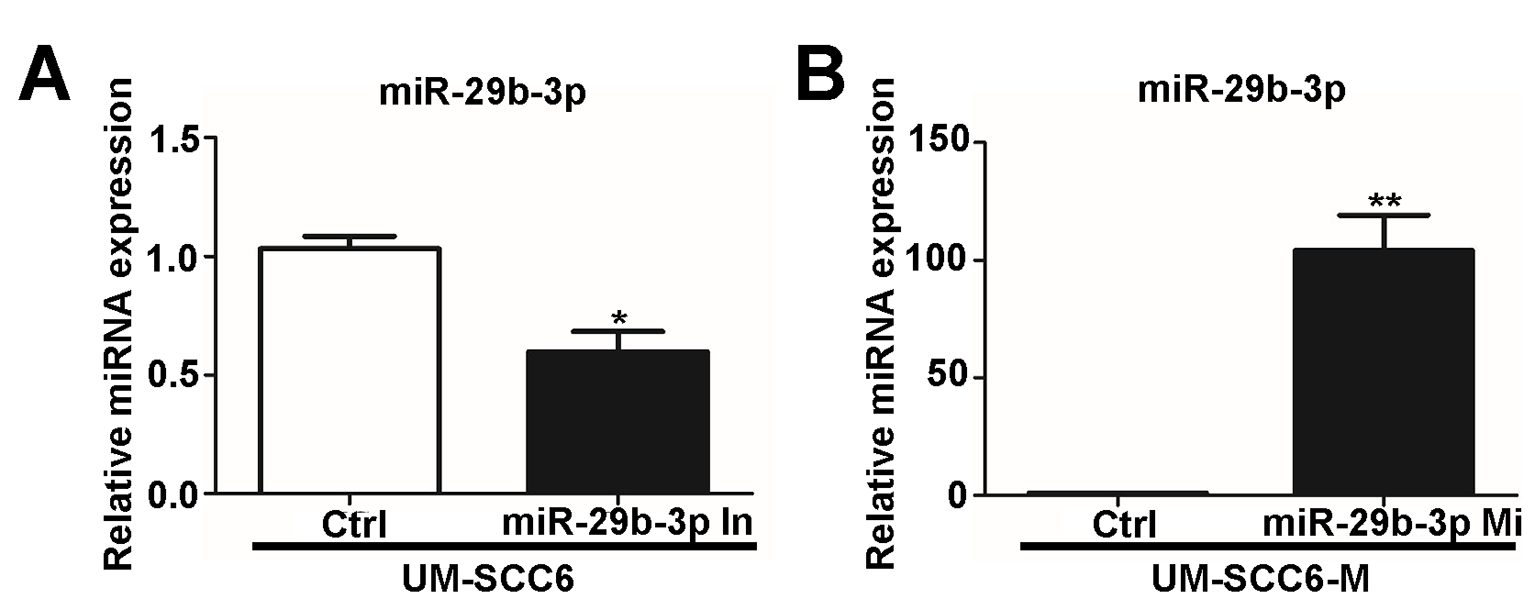

Supplement: Supplementary file 2 [file JCMM-24-841-s002.tif]

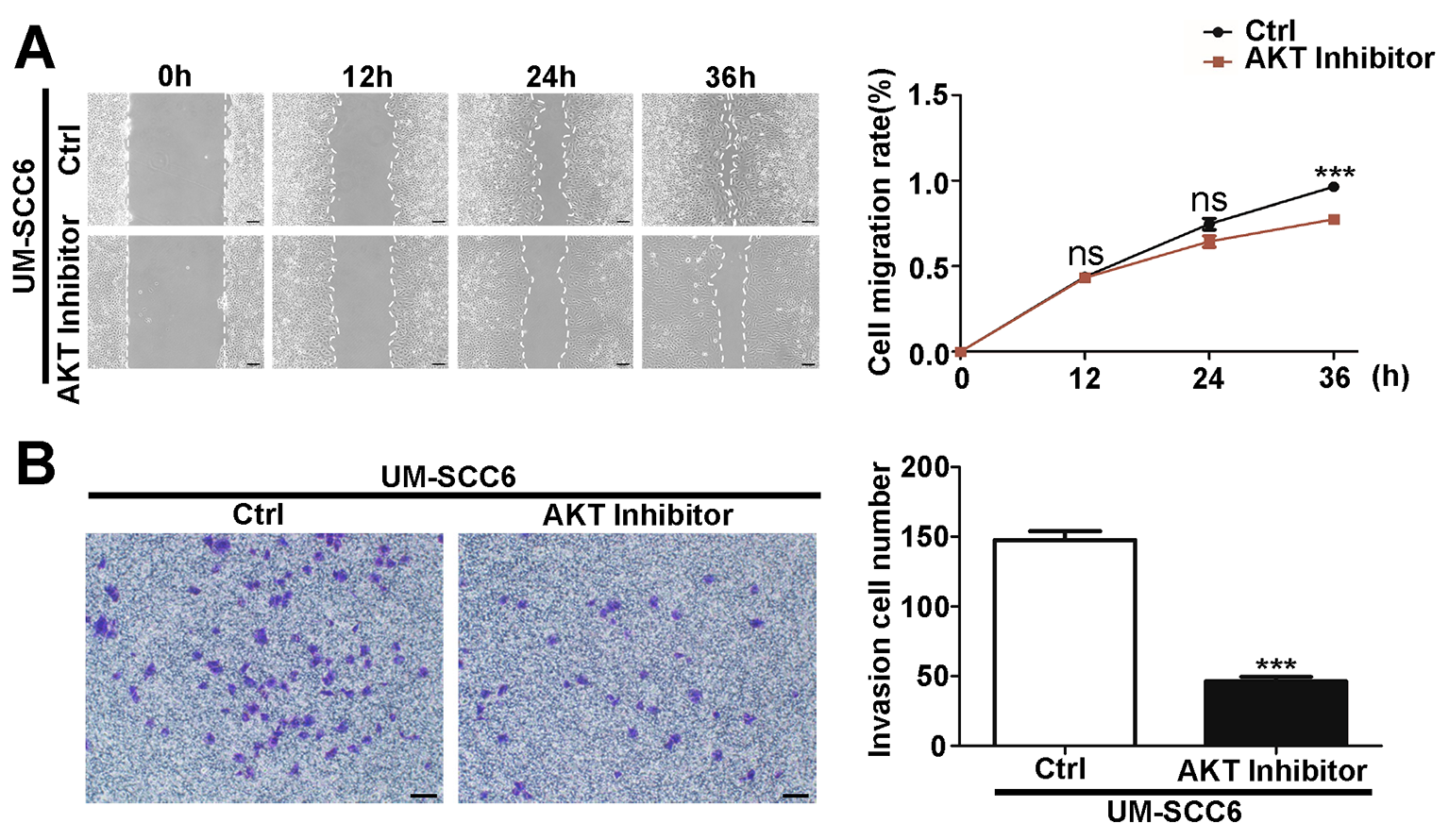

Supplement: Supplementary file 3 [file JCMM-24-841-s003.tif]

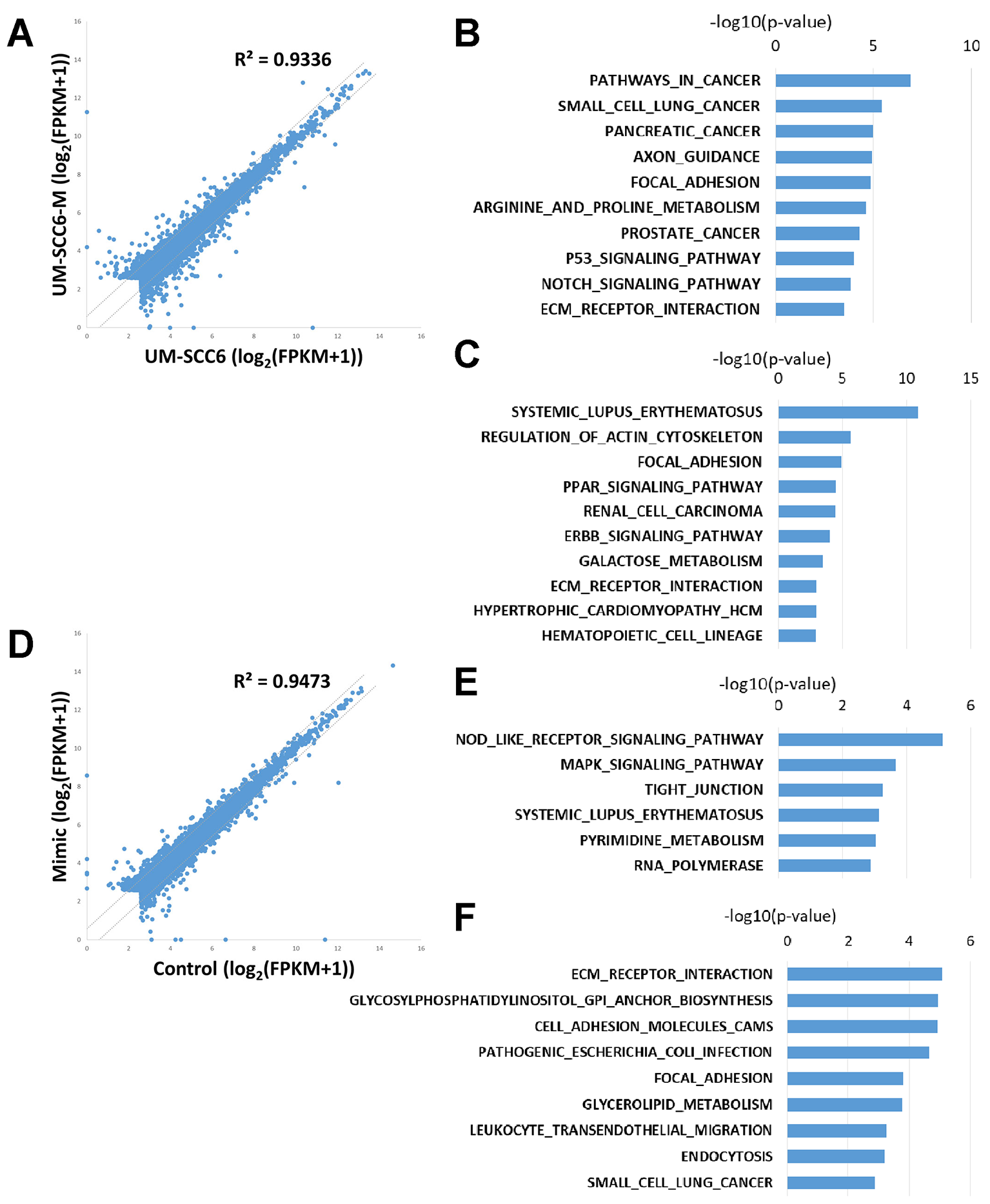

Supplement: Supplementary file 4 [file JCMM-24-841-s004.tif]

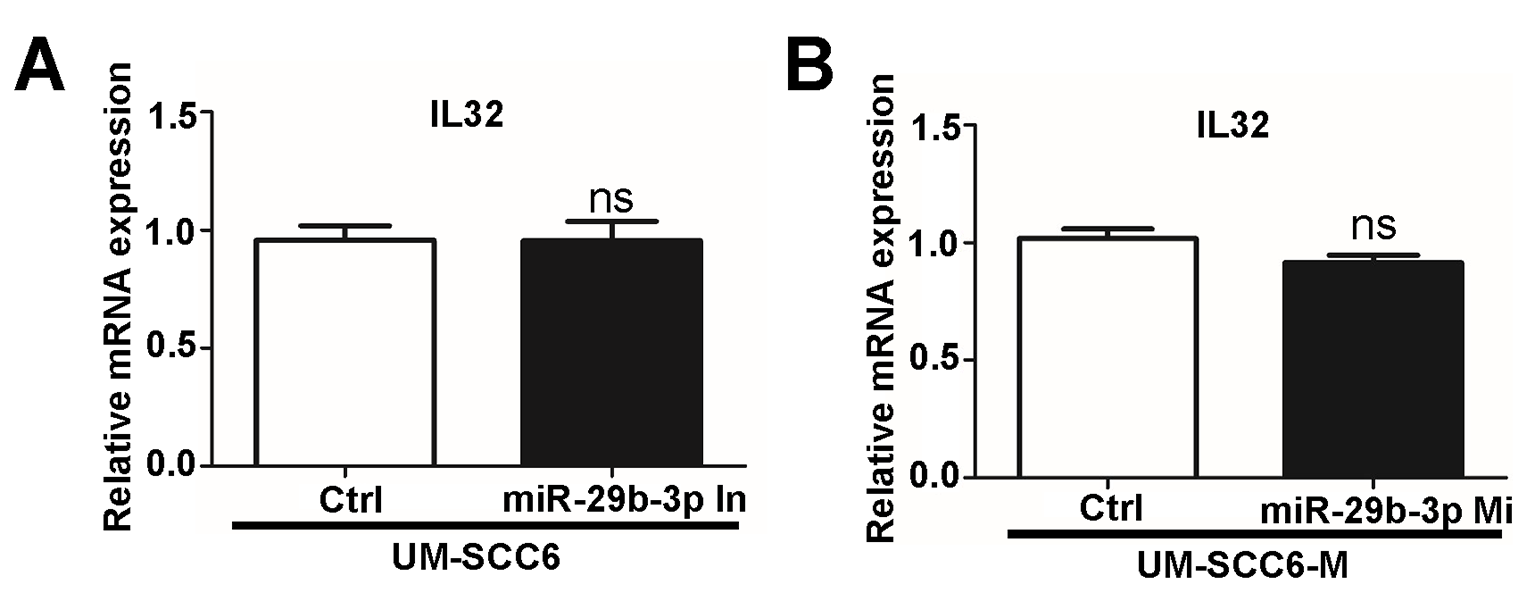

Supplement: Supplementary file 5 [file JCMM-24-841-s005.tif]

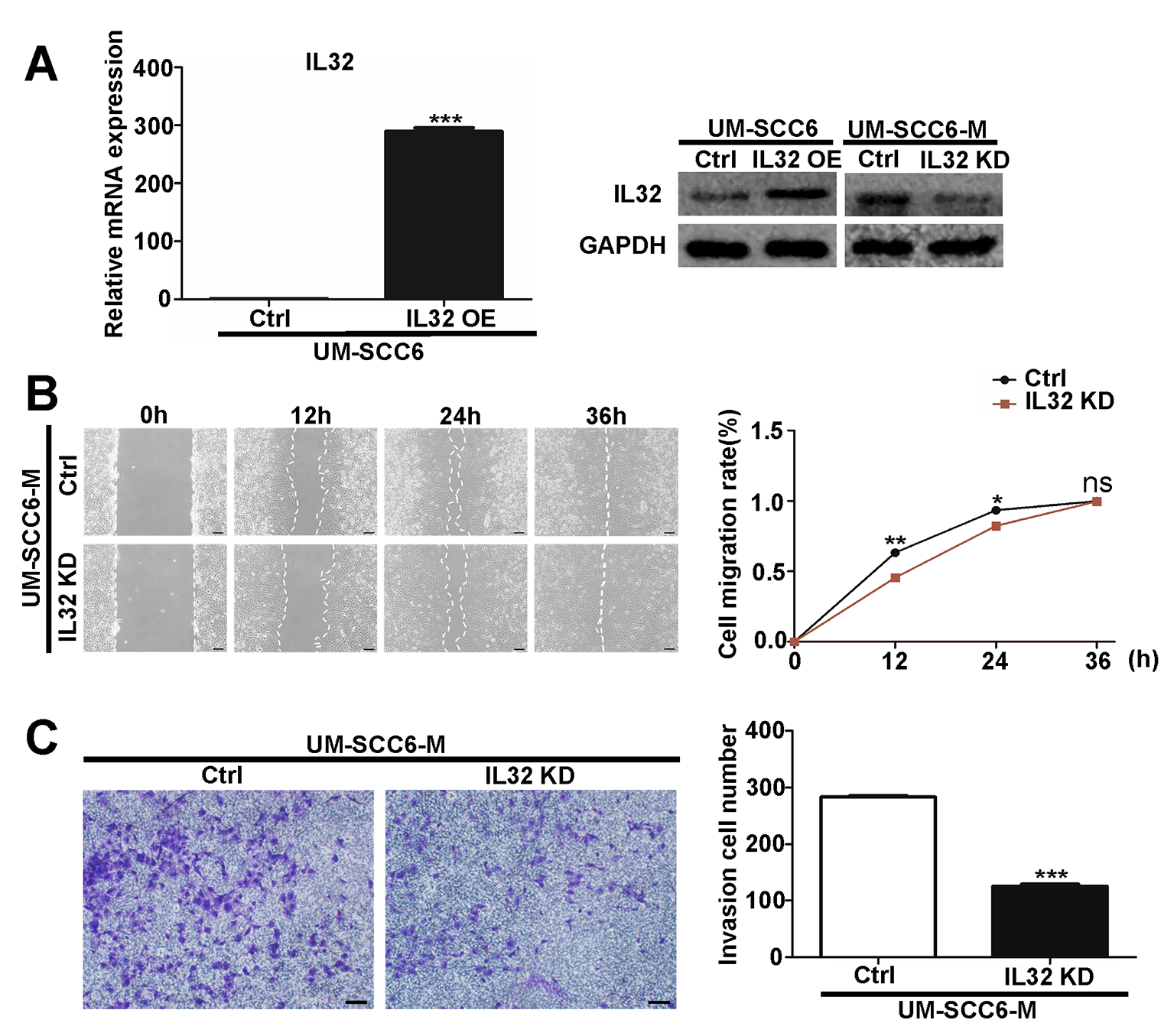

Supplement: Supplementary file 6 [file JCMM-24-841-s006.tif]

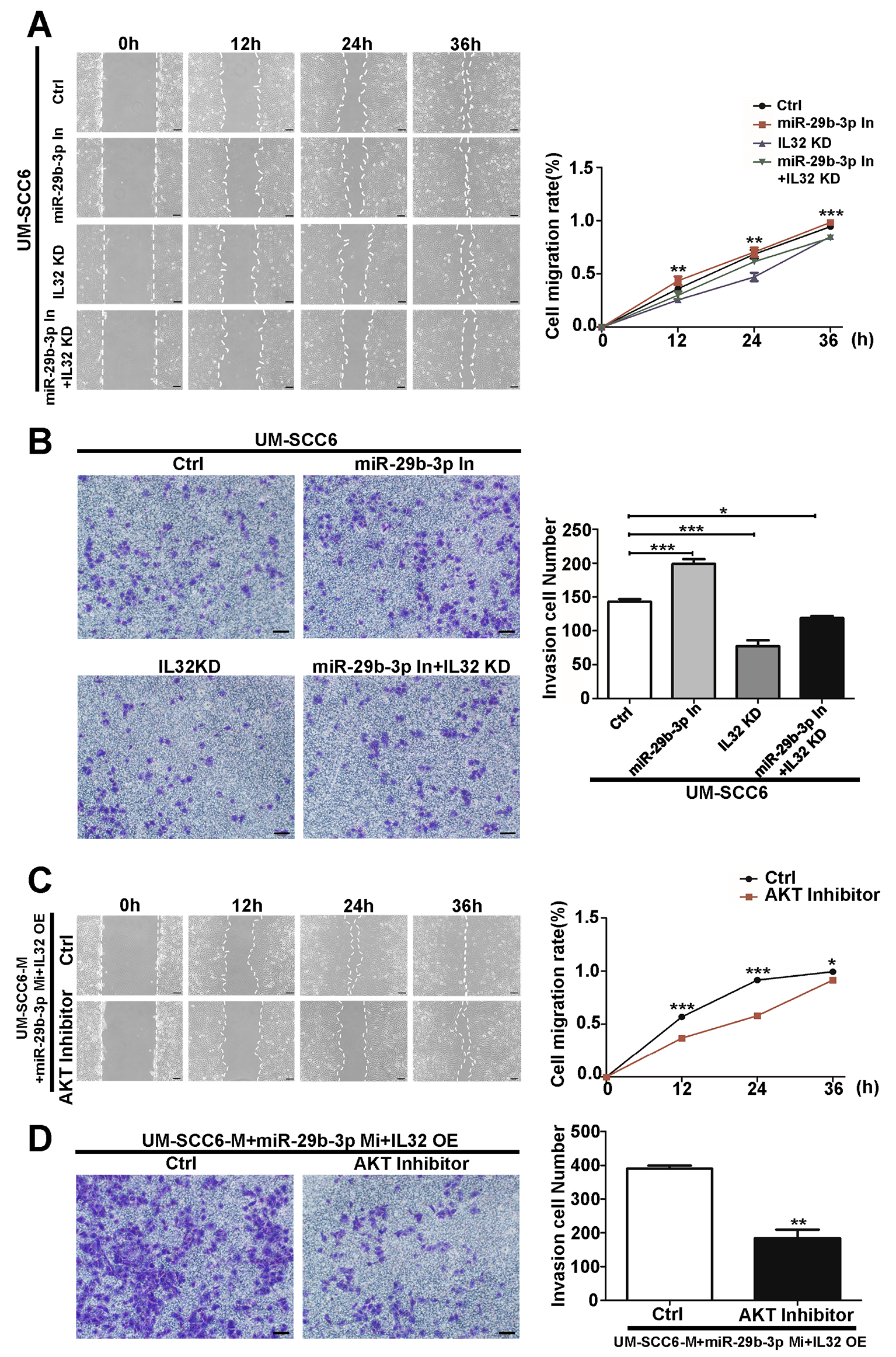

Supplement: Supplementary file 7 [file JCMM-24-841-s007.tif]
