# Supplementary material for: MicroRNA‐29b‐3p suppresses oral squamous cell carcinoma cell migration and invasion via IL32/AKT signalling pathway
Source: J Cell Mol Med. 2019 Nov 3;24(1):841–9. doi: 10.1111/jcmm.14794 (PMC6933408; doi:10.1111/jcmm.14794)
Supplement: Supplementary file 8 [file JCMM-24-841-s008.doc]

**Figure S1.** The morphology and migratory capacity of UM-SCC6 and UM-SCC6-M cells. **A**, Morphology of UM-SCC6 and UM-SCC6-M cells. **B**, Wound healing assay for UM-SCC6 and UM-SCC6-M cells. Scale Bar = 100 μm. Data represent the mean ± standard error of three independent experiments. **, P< 0.01; ns, not significant.

**Figure S2.** The effect of miR-29b-3p inhibitor and mimic. **A**, The RNA level of miR-29b-3p in UM-SCC6 after transfecting miR-29b-3p inhibitor and control. **B**, The RNA level of miR-29b-3p in UM-SCC6-M after transfecting miR-29b-3p mimic and control. Data represent the mean ± standard error of three independent experiments. *, P< 0.05; **, P< 0.01. In, inhibitor; Mi, mimic; Ctrl, control.

**Figure S3.** The effect of AKT signaling on the migration and invasion of UM-SCC6 cells. **A**, Wound healing assay showed the effect of MK-2206 on UM-SCC6 cells’ migration. **B**, Transwell invasion assay showed the effect of MK-2206 on UM-SCC6 cells’ invasion. Scale Bar = 100 μm. Data represent the mean ± standard error of three independent experiments. ***, P< 0.001; ns, not significant.

**Figure S4.** Transcriptional profile analysis. **A**, Scatter plots for genes expression profile in UM-SCC6 and UM-SCC6-M. **B** and **C,** Enriched KEGG pathways for upregulated genes in UM-SCC6 and UM-SCC6-M, respectively. **D**, Scatter plots for genes expression profile in UM-SCC6-M transfected with control and miR-29b-3p mimic. **E** and **F,** Enriched KEGG pathways for upregulated and downregulated genes with the transfection of miR-29b-3p mimic in UM-SCC6-M cells, respectively.

**Figure S5.** The influence of miR-29b-3p on IL32 at mRNA level. **A**, The RNA level of IL32 in UM-SCC6 after transfecting miR-29b-3p inhibitor. **B**, The RNA level of IL32 in UM-SCC6-M after transfecting miR-29b-3p mimic. Data represent the mean ± standard error of three independent experiments. ns, not significant.

**Figure S6.** The effect of IL32 on the migration and invasion of UM-SCC6-M cells. **A**, The RNA (left) and protein level of IL32 after transfecting IL32 overexpression and knockdown plasmids. **B**, Wound healing assay showed the effect of IL32 knockdown on migration ability of UM-SCC6-M cells. **C**, Transwell invasion assay showed the effect of IL32 knockdown on invasion ability of UM-SCC6-M cells. Scale Bar = 100 μm. Data represent the mean ± standard error of three independent experiments. *, P< 0.05; **, P< 0.01; ***, P< 0.001; ns, not significant. OE, overexpression; KD, knockdown.

**Figure S7.** miR-29b-3p suppressed migration and invasion via the IL32/AKT pathway. **A**, Wound healing assay showed IL32 knockdown attenuated the influence of miR-29b-3p inhibitor on migration of UM-SCC6 cells. **B**, Transwell invasion assay showed IL32 knockdown attenuated the influence of miR-29b-3p inhibitor on invasion in UM-SCC6 cells. **C**, **D**, Effect of MK-2206 on the migration and invasion of UM-SCC6-M cells co-transfected with IL32 overexpression plasmid and miR-29b-3p mimic, as evaluated by wound healing and transwell invasion assays, respectively. Scale Bar = 100 μm. Data represent the mean ± standard error of three independent experiments. *, P< 0.05; **, P< 0.01; ***, P< 0.001.
